# Disentangling the Spatio-Environmental Drivers of Human Settlement: An Eigenvector Based Variation Decomposition

**DOI:** 10.1371/journal.pone.0067726

**Published:** 2013-07-02

**Authors:** Ralf Vandam, Eva Kaptijn, Bram Vanschoenwinkel

**Affiliations:** 1 Sagalassos Archaeological Research Project, Faculty of Arts, University of Leuven, Leuven, Belgium; 2 Laboratory of Aquatic Ecology, Evolution and Conservation, University of Leuven, Leuven, Belgium; University of Oxford, United Kingdom

## Abstract

The relative importance of deterministic and stochastic processes driving patterns of human settlement remains controversial. A main reason for this is that disentangling the drivers of distributions and geographic clustering at different spatial scales is not straightforward and powerful analytical toolboxes able to deal with this type of data are largely deficient. Here we use a multivariate statistical framework originally developed in community ecology, to infer the relative importance of spatial and environmental drivers of human settlement. Using Moran’s eigenvector maps and a dataset of spatial variation in a set of relevant environmental variables we applied a variation partitioning procedure based on redundancy analysis models to assess the relative importance of spatial and environmental processes explaining settlement patterns. We applied this method on an archaeological dataset covering a 15 km^2^ area in SW Turkey spanning a time period of 8000 years from the Late Neolithic/Early Chalcolithic up to the Byzantine period. Variation partitioning revealed both significant unique and commonly explained effects of environmental and spatial variables. Land cover and water availability were the dominant environmental determinants of human settlement throughout the study period, supporting the theory of the presence of farming communities. Spatial clustering was mainly restricted to small spatial scales. Significant spatial clustering independent of environmental gradients was also detected which can be indicative of expansion into unsuitable areas or an unexpected absence in suitable areas which could be caused by dispersal limitation. Integrating historic settlement patterns as additional predictor variables resulted in more explained variation reflecting temporal autocorrelation in settlement locations.

## Introduction

Spatial correlation (i.e. a geographic dependency of observations) is a fundamental attribute of the organization of biological systems [1], [2] ranging from the growth of spatially discrete bacterial colonies in petri dishes over the patchy structure of plant and animal populations up to the spatial distribution of human settlements in the landscape [3]. Clustering of biological units such as individuals or populations can be driven by both environment dependent and - independent processes. Environments are typically heterogeneous at different spatial scales ranging from subtle differences in the physico-chemical environment of individual organisms up to large scale variation in habitat structure across landscapes driven by historic geomorphological processes and broad environmental gradients such as climate and productivity. Certain areas also typically contain more limiting resources than others or better meet the requirements of certain species than others [4]. As a result these localities are more likely to be colonized and sustain populations. This type of deterministic distribution of organisms in space based on the quality of the environment is known as environmental filtering or species sorting [5]. This paradigm, however, assumes that there are no restrictions in the movements of organisms and that ultimately all suitable patches will be occupied. In reality, this assumption is often not met as migration is often not that efficient and certain suitable patches will remain unoccupied: a pattern known as dispersal limitation [6]. Individuals and populations can also be unable or unwilling to migrate and resettle even though local conditions are no longer suitable [7]. While in animals and plants inability to migrate is a main reason why suitable patches remain unoccupied, social, cultural, financial and political barriers might play a similar role in humans [8]. Additionally, species sometimes expand into unsuitable areas where they would normally go extinct but nonetheless manage to persist as a result of continuous arrival of new migrants from sources (source sink dynamics) [9], [10]. Examples of this in human societies include, for instance, villages or a city such as Ancient Rome that cannot sustain themselves and would perish if resources and people were not continuously brought in from other sources through exchange or trade. Finally, historical patterns such as the point of entry of a species or race in a region or the location of the first population or settlement can have important consequences for the expansion of a species into an area resulting in persisting founder effects [11], [12]. Consequently, in the case of time series analyses it can be feasible to use historic distributions as predictors of later patterns (temporal autocorrelation [13]). Disentangling the relative importance of history, environmental and spatial processes explaining distribution patterns, however, remains an important challenge both in ecology [14], anthropology and archaeology [15], [16], [17]. In general, analyses of ancient settlement patterns typically conclude by pointing out a single environmental variable of presumed importance [15] without rigorously assessing the explanatory value of different sets of variables explaining reality [17]. Different statistical approaches have been developed that can take into account the spatial structure of sites such as classical isolation by distance analysis using intuitive but limited partial Mantel tests [18], [19] or multivariate ordinations including spatial descriptors such as polynomials constructed from X and Y coordinates (trend surface analysis [20]). The recent development of advanced spatial descriptors (PCNM - principal coordinates of neighboring matrices; MEM – Moran’s eigenvector maps [21], [22], [23]), however, provides important new opportunities since these are more powerful at detecting spatial variation and allow to identify the scales at which spatial clustering occurs. This is important since aggregation might be beneficial at certain scales but detrimental at others. For instance, while people may be more inclined to settle close to other settlements because they can trade with them or because of the abundance of a certain resource, starting up a new settlement too close to existing settlements may be a bad choice as it can lead to social-economic problems such as resource competition which may lead to conflict [24] or abandonment of sites and shifts in settlement patterns [15]. What is more, by including sets of predictor variables representing spatial variables as surrogates for migration-based processes, environmental variables indicative of environmental filtering and historic factors in multivariate ordination models it is possible to use a variation partitioning procedure [25] to separate the unique effects of different sets of variables as well as the variation that is commonly explained by different variation components. For instance, the effects of an environmental condition that is confined to a certain geographic area may be detected as spatially structured environment. As such, the method is able to distinguish whether spatial distribution patterns are the result of spatially clustered environmental conditions or environment independent processes such as source sink dynamics or dispersal limitation [26]. While this method has been extensively used in ecology, its potential use in other disciplines such as the social sciences remains largely unexplored.

We applied this method on a dataset of archaeological artefacts collected in a region in south western Turkey and spanning a time period of almost 8000 years from the Late Neolithic/Early Chalcolithic (6500–5500 BC) up to the Byzantine period (610–1300 AD). We investigate changes in the relative importance of history (presence of earlier settlements), environmental and spatial variables explaining settlement distributions over time and discuss the relative importance of different processes that can explain clustering of human populations at different spatial scales. For this the dataset was subdivided into six periods which could be reliably distinguished based on period-characteristic material culture (1: Late Neolithic/Early Chalcolithic (6500–5500 BC); 2: Late Chalcolithic/Early Bronze Age I (4000–2600 BC); 3: Early Bronze Age II (2600–2300 BC); 4: Archaic-Classical/Hellenistic (750–200 BC). 5: Hellenistic (333–25 BC); 6: Byzantine (610–1300 BC).

## Methods

### Ethics Statement

The research permit was granted by the Turkish Ministry of Culture and Tourism, General Directorate of Cultural Properties and Museum. All necessary permits were obtained for the described study, which complied with all relevant regulations.

### Study Area

**Figure 1:**
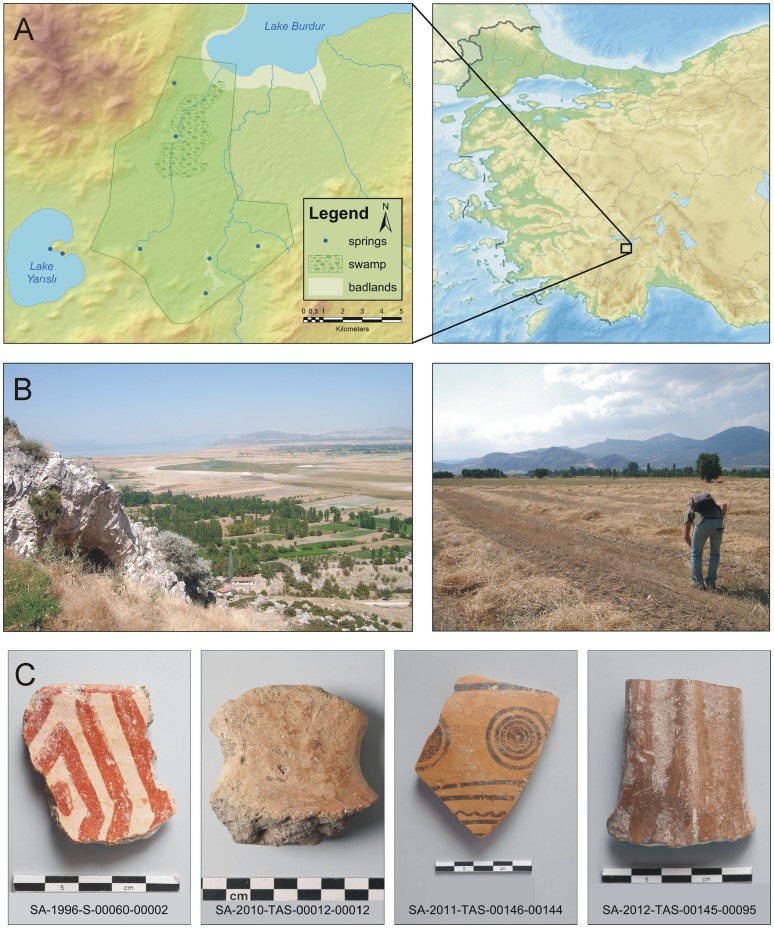
Background of the research area. Location of the research area (A) in the Burdur plain situated in the lake district of south-western Turkey. Blue dots represent springs (B) Overview picture of the Burdur plain featuring Lake Burdur in the background and typical field conditions in the valley plain during surveys. (C) Examples of pottery fragments indicative for different time periods. In chronological order from left to right: Early Chalcolithic, Late Chalcolithic, Archaic-Classical Hellenistic and Byzantine periods.

The research area consists of the southern part of the Burdur plain which is located in the Turkish Lake District and surrounded by the western Taurus Mountains (Fig. 1). It is situated in a tectonic ‘graben’ system [27], [28] of which the central part is occupied by Lake Burdur. The level of this lake has fluctuated considerably during the lake’s history, however. Around 20 000 BP, the lake reached its highest level and has declined ever since [29]. The retreating lake resulted in a flat plain and the lacustrine deposits provided fertile soils suitable for agriculture. Two rivers drain the southern part of the Burdur plain, the Duğer Çayı and the Boz Çayı. From an archaeological point of view, the Burdur plain is considered as an important area. Previous excavations at Hacilar [30], Kuruçay Höyük [31], [32] and the ongoing excavation of the University of Istanbul at Hacilar Büyük Höyük [33] have revealed unique information on the Late Neolithic, Chalcolithic and Early Bronze Age periods in Anatolia. These excavations make the Burdur Plain one of the best studied regions of Anatolia for these time periods and even beyond. However, little is known about other possible settlements in the vicinity of these excavated sites. To remedy this, the Sagalassos Archaeological Research Project started a series of intensive survey seasons in the Burdur plain in 2010, which resulted in the discovery of hamlets and farmsteads dating from Late Prehistoric to Ottoman times [34], [35].

### Data Collection

The area was surveyed by a team of researchers walking transects of 50×1 m spaced 20 m apart and collecting all manmade artefacts predating the 1920’s in the landscape. For the present analysis, the study area was divided in a regular grid of 9986 cells of 90×90 m. Based on GPS coordinates, artefacts collected in transects were assigned to corresponding grid cells in the dataset. From the collected artefacts only those that could be reliably attributed to a certain time period were retained for further analyses. Although absolute synchronicity of sites cannot be attested via the relative dating of archaeological survey, the fact that identical pottery fabrics, probably stemming from a single production center, were used on sites from the same time period suggests synchronicity (unpublished data). An overview of different artefacts and the time periods to which they correspond is provided in Table S1. In order to improve the resolution of the dataset it was decided not to simply attribute artefacts to certain grid cells based on the exact geographic location (all or nothing principle) falling within a certain cell. Instead, we used a simple weighting method based on the distance of each artefact to the four nearest grid cell centroids according to (Eq. 1). In this formula *A_xy_* represents the artefact abundance assigned to a certain grid cell A with centroid coordinates x and y. N = the number of artefacts found in this cell, ai = the Euclidean distance of an artefact in cell A to the centroid of that cell and bi, ci and di = the Euclidean distances to the other three nearest cell centroids. As such, artefact abundance (calculated separately for artefacts from each time period) of each grid cell is no longer an integer number, but the column sum still correctly represents the total number of artefacts collected in the area. This approach can be considered as an elegant way to smoothen the response data, reducing the importance of the exact location of individual artefacts, which often will have been moved due to localized disturbances such as plowing. Using this approach a site x artefact abundance response datamatrix was constructed with six columns corresponding to each of the considered time periods.
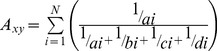
(1)


### Environmental Data

Environmental properties of each cell (the unit of the analysis) were attributed to the cell’s centroid and included elevation (m asl), distances to the nearest river, spring and hill and the percentage of different land cover types in radii of one and four km, respectively. Considered land cover categories included hills, badlands, valley floor, swamp and lake, as reconstructed from available GIS data layers. A last variable represents the estimated visible area of an object of 1 m high for an observer of 1.50 m and assuming a maximum visibility of 10 km. A detailed overview of the 15 environmental variables considered in this study is presented in Table S2.

### Data Analysis

The distribution of archaeological artefacts from each of the six considered time periods was analysed using a variation partitioning procedure [25], [36] based on redundancy analysis (RDA) models [23] with significances tested using 999 Monte Carlo permutations. This procedure decomposes the total variation in the response dataset into a pure spatial component (S|E), a pure environmental component (E|S), a component of the spatial structured environmental variation (E∩S) and the unexplained variation. Only significant predictor variables identified using a forward selection procedure [37] based on the adjusted R^2^ stopping criterion were retained in constructed models [38]. Artefact abundances were Hellinger transformed [39] prior to analyses (divide the abundances by the row sum and take the square root of the resulting ratiosartefact).

In order to analyse the importance of spatial autocorrelation at different spatial scales, a set of Moran’s Eigenvector Maps (MEM’s) was constructed [21], [40]. In a regular matrix of sites these variables are wave-functions with different wave lengths corresponding to spatial correlation at different spatial scales [22], [40] (Fig. S1). Only MEM’s that have significant positive spatial autocorrelation as calculated using Moran’s I [41] were used in the analyses. Forward selection was performed on this set of eigenfunctions. Only significant MEM variables retained in constructed models for each time period were included. Analogous to the variation partitioning procedure outlined above, spatio-environmental covariation was corrected for by including significant environmental variables as covariables in these analyses [23].

Secondly, in order to assess the potential importance of existing settlements during the previous time period as determinants of current settlement patterns, the artefact abundances in time period T minus1 were included as an additional variation component history [H] besides space [S] and environment [E] in a second set of variation partitioning analyses.

Finally, by analyzing the fit of all MEM variables corresponding to significant positive autocorrelation with response variables of interest it is possible to investigate at which spatial scales, spatial clustering occurs in the dataset. Frequency distributions of the wavelengths (λ) of the MEM variables retained in RDA models after forward selection are generated in order to assess variation in the scales of spatial clustering that are relevant during different time periods. Wavelength is expressed in km and for a regular grid it can be interpreted as the distance between the centers of neighboring clusters. To test the ability of MEM’s with increasing wavelengths in explaining observed variation in artefact abundances, the amount of variation (adjusted R^2^) explained by pure spatial variation (S|E) was calculated for consecutive sets 10 MEM’s corresponding to increasing spatial scales. Additionally, the fitted site scores of the dominant first canonical axis of RDA models explaining the abundance of archeological artefacts using MEMs are plotted to highlight areas where artefact distributions are successfully predicted by MEM predictors.

All analyses were carried out in R version 2.15.0 (R Development Core Team 2012) using the packages **PCNM** (MEM variables), **AEM** (Moran’s I spatial autocorrelation), **vegan** (Hellinger transformations, RDA, variation partitioning) and **packfor** (forward selection).

## Results

**Figure 2:**
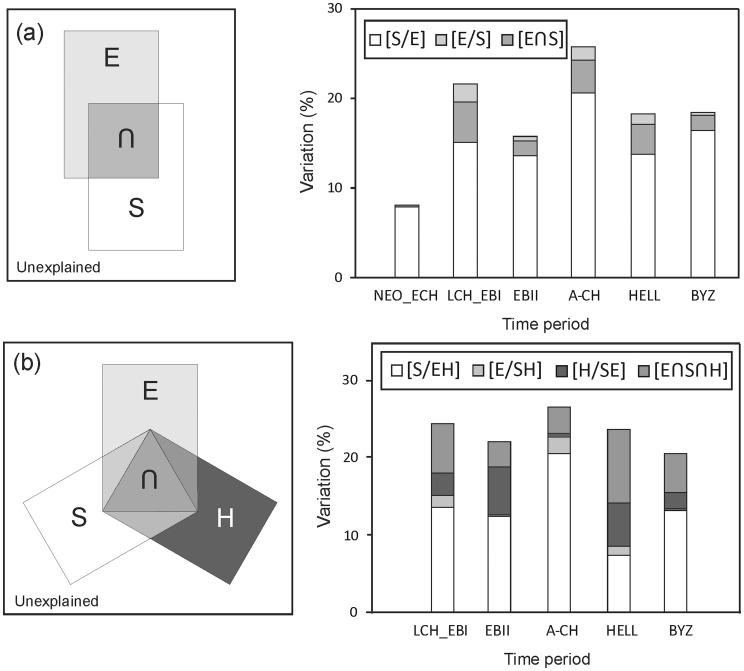
Variation partitioning showing the amount of variation in human settlement patterns explained by different variation components. The upper panel (a) depicts results of a partitioning of variation based on spatial and environmental variables. The lower panel (b) shows results of a partitioning of variation based on a spatial, environmental and historical variation component. The historical variation component corresponds to artefact abundance patterns during the preceding time period. [E/S] = pure environmental variation corrected for space, [E/SH] = pure environmental variation corrected for space and history, [S/E] pure spatial variation corrected for environment, [E/SH] = pure spatial variation corrected for environment and history, [H/SH] = pure history, corrected for space and environment, [E∩S] = variation commonly explained by space and environment, [E∩S∩H] = variation commonly explained by space, environment and history.

The first 5249 MEM’s had positive eigenvalues. Of these only 1215 were characterized by significant positive spatial autocorrelation based on Moran’s I and were consequently retained in further analyses. Overall RDA models predicted a substantial fraction of observed variation ranging between 8 and 26%. Variation partitioning revealed that both spatial and environmental variables explained a significant proportion of variation in our datasets, even after correction for collinearity with other variation components. Considering artefact abundance during the previous time period as a separate historical variation component [H] generally resulted in better models explaining more variation, except for time period A-CH (Table 1, Fig. 2).

**Table 1:** Variation partitioning of artefact abundance datamatrices for each of the six considered time periods with corresponding P values.

		T1		T2		T3		T4		T5		T6	
	Period	NEO_ECH		LCH_EBI		EBII		A-CH		HELL		BYZ	
		Var %	P	Var %	P	Var %	P	Var %	P	Var %	P	Var %	P
**(a)**	**[EUS]**	8.13	0.005	21.48	0.001	15.7	0.001	25.8	0.001	18.27	0.001	18.41	0.001
	**[E]**	0.25	0.005	6.5	0.001	2.20	0.001	5.10	0.001	4.67	0.001	2.18	0.001
	**[S]**	8.10	0.005	19.6	0.001	15.3	0.001	24.25	0.001	17.07	0.001	18.09	0.001
	**[E/S]**	0.04	0.03	1.88	0.001	0.45	0.001	1.56	0.001	1.21	0.001	0.32	0.001
	**[S/E]**	7.90	0.005	15.01	0.001	13.53	0.001	20.63	0.001	13.68	0.001	16.41	0.001
	**[E∩S]**	0.20		4.59		1.77		3.62		3.38		1.68	
	**1-[EUS]**	91.86		78.5		84.2		74.16		81.7		81.6	
**(b)**	**[EUSUH]**			24.25	0.001	21.9	0.001	25.80	0.001	23.5	0.001	20.49	0.001
	**[E]**			6.5	0.001	2.20	0.001	5.1	0.001	4.67	0.001	2.18	0.001
	**[S]**			19.6	0.001	15.3	0.001	24.25	0.001	17.07	0.001	18.09	0.001
	**[H]**			5.41	0.001	8.4	0.001	0.025	0.067	14.13	0.001	6.14	0.001
	**[E/SH]**			1.66	0.001	0.17	0.001	1.99	0.001	1.06	0.001	0.23	0.001
	**[S/HE]**			13.4	0.001	12.30	0.001	20.5	0.001	7.4	0.001	13.15	0.001
	**[H/SE]**			2.77	0.001	6.14	0.001	0	0.452	5.57	0.001	2.08	0.001
	**1- [EUSUH]**			75.75		78.09		74.2		76.5		79.51	

Presented variation estimates correspond to adjusted R^2^ values. Upper panel (a) represents results from a partitioning of spatial [S] and environmental variation [E]. In the lower panel (b), the presence of historic settlements present in the preceding time period [H] is included as an additional component in the analyses. NEO_ECH: Late Neolithic/Early Chalcolithic (6500–5500 BC); LCH_EBI: Late Chalcolithic/Early Bronze Age I (4000–2600 BC); EBII: Early Bronze Age II (2600–2300 BC); A-CH: Archaic-Classical/Hellenistic (750–200 BC). HELL: Hellenistic (333–25 BC); BYZ: Byzantine (610–1300 BC).

**Figure 3:**
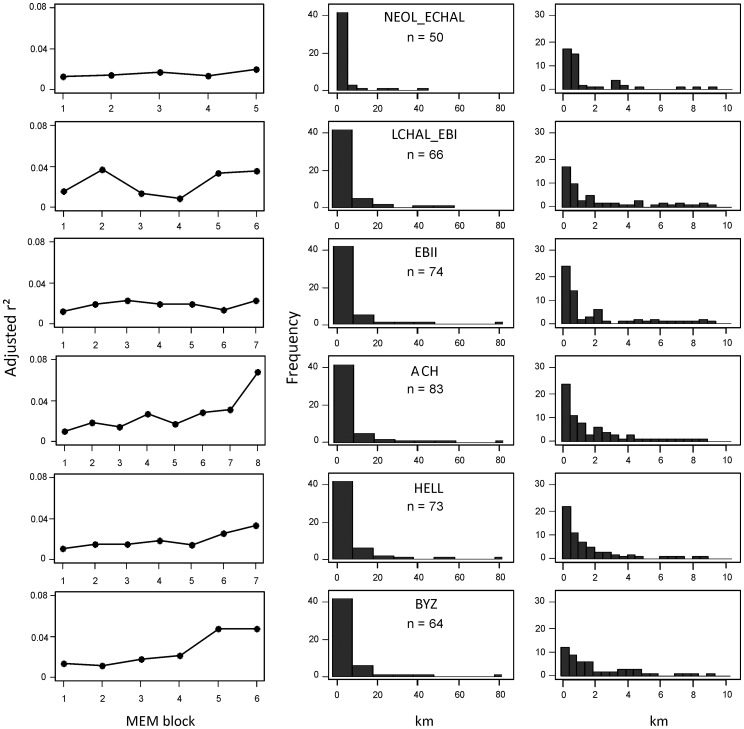
Variation in artefact abundances explained by sets of MEM variables corresponding to increasingly broader spatial scales. Left panel- Variation in artefact abundances (adjusted R^2^) explained by sets of MEM variables (10 variables per category) corresponding to increasingly higher spatial scales (right panel). Category one groups the ten significant MEM’s corresponding to the smallest λ, category two groups the following 10 MEM’s and so on, R^2^ was calculated using partial RDA models correcting for significant environmental variables and thus representing pure spatial variation [S/E]. Middle and right panel - frequency distributions of the wavelength (λ) of MEM variables retained in RDA models after forward selection illustrating variation in the scales of spatial clustering relevant during different time periods. Middle panels show all MEM’s, right panels zoom in on variation in spatial correlation within 0–10 km.

**Figure 4:**
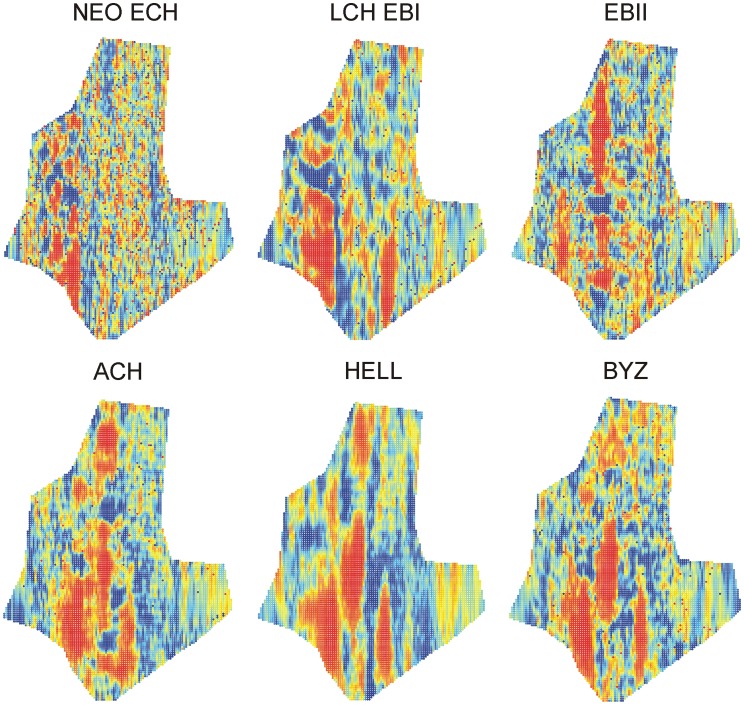
Maps of fitted site scores of the first canonical axis of RDA models explaining the abundance of archeological artefacts. Predictor variables are the significant Moran’s eigenvector maps retained after forward selection. A red color indicates high values, blue areas are indicative of low values. Separate maps are provided for RDA models for each of the six considered time periods. NEO_ECH: Late Neolithic/Early Chalcolithic (6500–5500 BC); LCH_EBI: Late Chalcolithic/Early Bronze Age I (4000–2600 BC); EBII: Early Bronze Age II (2600–2300 BC); A-CH: Archaic-Classical/Hellenistic (750–200 BC). HELL: Hellenistic (333–25 BC); BYZ: Byzantine (610–1300 BC).

In general, a higher abundance of artefacts was found in valleys and in closer proximity to springs and hills. The prevalence of swamp, in turn, had a negative effect on artefact abundance (Table 2). Significant spatial variables retained in our models included latitude and longitude, in general supporting a higher abundance of artefacts in the south-western corner of the area, which contains many hills and springs. MEM’s fitted a broad range of scales of spatial clustering with wavelengths (λ) ranging from 150 m to almost 90 km and there were no consistent differences between the considered time periods (mean±SD: NEO_ECH: 2.52±6.22 km; LCH_EBI: 4.1±10.7 km; EBII: 2.2±5.0 km; A-CH: 4.1±10.4 km; HELL: 3.3±9.5 km; BYZ: 3.8±10.9 km; Table S3). Overall, however, most MEM’s retained after forward selection corresponded to clustering with relatively small inter cluster distances varying between 100 m and 5 km (Fig. 3). Despite the higher abundance of small scale MEM’s (λ <2 km) retained after forward selection, partial redundancy analyses correcting for significant environmental variation, showed that sets of the larger scale MEM’s retained after forward selection typically explained more variation than sets of smaller scale MEM’s and this effect was most pronounced in later time periods (Fig. 3). Similarly, maps showing the fit of the first canonical axis to artefact abundance data, also show larger spatial clusters of cells where artefact abundances are adequately predicted by MEM variables in later time periods (Fig. 4).

**Table 2:** List of significant environmental and spatial predictor variables retained after a forward selection procedure in RDA models explaining the abundance of archaeological artefacts from each of the six considered time periods.

Period		T1	T2	T3	T4	T5	T6
		NEO_ECH	LCH_EBI	EBII	A-CH	HELL	BYZ
Env.	NEAR_SPRNG	+	+	+	+	+	
	NEAR_HILL		+		+		+
	NEAR_RIVER				+	+	
	P_VALLEY1		+		+	+	
	P_VALLEY4		+		+	+	+
	P_SWAMP1			**–**	**–**	**–**	**–**
	P_SWAMP4		**–**	**–**			**–**
	P_BADLAND1			**–**	**–**	**–**	
	P_BADLAND4		**–**	**–**	**–**		**–**
	P_HILL1				**–**	**–**	
	P_HILL4				**–**		
	P_LAKE1				**–**	**–**	
	P_LAKE4				**–**		
	ELEVATION			**–**	**–**	**–**	
	VIEWSHED_1.4		**–**	**–**	**–**		**–**
Space	Coordinates	X. Y	X. Y	X	X. Y	X. Y	X.Y
	No. sign. MEMs	50	66	74	83	73	64

(+) and (**–**) designate positive and negative effects. respectively. The variables NEAR_SPRNG. NEAR_HILL and NEAR_RIVER i.e. the distance to the nearest river, hill or lake were included in models as distances. As a result the sign of the measured effect was inversed in order to be able to interpret these variables as proximities.

## Discussion

Results illustrate how patterns of human settlement can be decomposed into spatial correlation at different spatial scales. Part of this correlation was shown to be caused by spatially structured environmental conditions while most spatial correlation was independent of environmental gradients considered in our analyses. As outlined in detail below, these results reflect a number of general processes generating spatial structure in human societies. Additionally, despite the incompleteness of the archaeological record these relatively simple models considering a relatively small set of environmental variables already explained up to 26% of observed variation in artefact abundances. This indicates that human settlement in this region was characterized by a relatively strong deterministic component.

### Environmental Filtering

Our results, first of all, showed clear significant links between settlement patterns and local environmental conditions. In nature different processes can result in a close match between environmental gradients and species distributions. For many organisms that cannot control their own migration such as plants and many invertebrates, environmental filtering occurs as a result of random dispersal followed by differential establishment success [42]. Other groups and particularly higher organisms typically have better dispersal abilities and more complex nervous systems enabling them to use environmental cues to determine whether a locality is suitable for settlement: a process known as active habitat selection [43]. The combination of superior dispersal ability and the intellectual ability to make rational decisions makes that particularly the latter will probably be the most prominent process driving differential human settlement in environmentally heterogeneous landscapes, as observed in this study. At larger spatial scales and when suitable areas are in scarce supply, however, it is likely that human settlement may also reflect random dispersal followed by differential mortality. A potential example of this could be the colonisation of islands in the isolated parts of the Pacific Ocean by seafaring Polynesian people [44]. Examples of environmental filtering emerging from this study include preferential settlement close to water sources (springs, rivers, lakes), hilltop lookouts and in lowland valleys, as also reported for settlements other regions of the Near East and the Aegean [45]. The availability of water appears to have been important during the entire history of the area, which is logical as water is indispensable for settlements and especially for (early) farming communities and their settlement location choice [46]. In contrast, a significant effect of the abundance of lowland valley around settlements was only detected from the Late Chalcolithic-Early Bronze Age onwards. There are solid indications that agriculture was already practiced in the area for about 1000 years prior to this time period [47], [48]. Yet, it is very likely that higher populations sizes and an increase in the number of settlements from that time onward can explain why the predominant settlement in valleys is only detected in our dataset since the Late Chalcolithic-Early Bronze Age due to higher statistical power. Abundance of good land for agriculture, however, may not have been the only motivation for settling in valleys as other advantages such as the availability of resources such as good hunting grounds, wild plants, clay sources [49], [50] or mobility and communication [51] may have been important. Areas dominated by badlands and marshland seem to have been avoided. While swamps may offer good hunting grounds for waterfowl as seen during the Neolithic period of Çatalhöyük [52] and could be used for collecting wetland plants [53], this type of land is unsuitable for agriculture and has the additional disadvantage that it can act as an important source of disease vectors such as mosquitoes transmitting malaria and other diseases [54], [55].

### Spatial Processes

Besides environmental filtering, our analyses show clear evidence of spatial clustering independent of the considered environmental variables. In fact, the majority of explained variation was described by pure spatial variation. A similar observation was made for the Early Neolithic settlement pattern in Thessaly, Greece [56]. This pattern may arise due to different processes. First of all, it can be an important indication for expansion of settlements into areas which, according to our models, would be deemed suboptimal and even unsuitable in terms of environmental conditions linked to resources. It is possible that these settled areas were not self sustaining and persisted by importing resources from elsewhere. This, however, is contradicted by current ideas on human settlements at that time which are assumed to be self supporting [57]. The presence of sites under suboptimal environmental conditions might point towards a non-domestic/non-agricultural function of these sites as sanctuaries [58], cemeteries [59], artisanal workshops [60] or even temporary camps for transhumant herdsman[61]. Secondly, individuals might also choose not to settle in presumed optimal areas close to certain critical resources if this means that they are further away from other resources. Under such conditions it could be more efficient to settle in apparently suboptimal localities that are situated at reasonable distances from a set of resources [50]. Thirdly, spatial grouping of settlements can be promoted because of inherent benefits such as increased protection, better risk management in times of crop failure when communities can rely on one another [62], improved interactions and exchange of knowledge and goods [63] and, lastly, social advantages such as the establishment of marriage networks [64], [65] and the opportunity to engage in social gatherings and communal events like feasting [66], [67]. Finally, spatial clustering may be associated with environmental characteristics that were not or could not be considered in this study. This scenario is likely since a considerable amount of relevant environmental information for human settlement during the considered time periods cannot be inferred based on recent observations. Current knowledge on historic conditions is notoriously incomplete and, as a result, it is possible that, for instance, environmental conditions that used to be spatially clustered in the past will now be described by spatial variables and detected as pure spatial variation.

Spatial variables were selected describing both large and small scale correlation. In general artefacts were typically clustered at scales smaller than 1–2 km with 75% of the MEM spatial descriptors that were retained after forward selection corresponding to correlation at scales <2 km. This indicates that typical inter-settlement distances were probably larger than this threshold ranging from anywhere between 1 and several km, a distance which matches well with commonly accepted estimates of the distance that can be covered while walking during one day [68]. Nonetheless, despite this, sequential analysis of the variation in the response dataset explained by blocks of 10 MEMs corresponding to increasing spatial scales revealed higher levels of explained variation by large scale MEM’s (typically >20 km) even when correcting for environmental variation. Aggregation at this scale could result from different processes including the outward spread of a population from a certain centre of origin [12] or can be the result of the presence of certain unknown resources as discussed higher up.

### Spatio-environmental Covariation

While we found unique effects of environmental conditions, independent of spatial variables [E] as well as unique effects of spatial correlation independent of similarities in environmental conditions [S], some variation was explained by spatio-environmental covariation, i.e. the variation that is explained jointly by space and environment [S***∩***E]. As a result, one cannot unequivocally attribute this explained variation to spatial or environmental processes. Often, environmental conditions themselves will be clustered in space resulting in a correlation between environmental similarity and spatial proximity, which is captured by the [S***∩***E] component in the analyses. In the current study this component seems to be less important than pure spatial variation but more important than pure environmental variation. Such a pattern was anticipated since particularly at larger spatial scales environmentally similar conditions tend to be spatially clustered [26].

### Historic Factors

Overall, our results not only reflect spatial but also temporal autocorrelation. In the absence of strong environmental change or important demographic events such as disease outbreaks or wars, it is logical that suitable localities will remain settled throughout the history of an area. Additionally, social and ideological elements such as ancestral worship and location bound ideologies [50], [51] can contribute to persistence of settlements in the same location. Indeed, artefact data suggested that settlement patterns during preceding time periods were generally a good indicator of settlement patterns in the different time periods considered in this study. What is more, taking historic patterns into account led to models that explained up to 6% more variation in settlement patterns than models that did not take this into account. For most considered time periods history had a significant unique effect on settlement patterns, both independent of spatial and environmental variation. This does not hold true for the A-CH period. This discrepancy is due to a chronological gap in the dataset just before this period, as artefacts from the Middle to Late Bronze Ages as well as from Early Iron Age were very scarce probably reflecting a very low level of human activity in the area. Finally, since ultimately earlier settlement patterns are affected by space and environment (resulting in autocorrelation) it is no surprise that a substantial proportion of variation [E**∩**S**∩**H] was jointly explained by space, environment and history.

### Stochasticity and Unexplained Variation

The large proportion of unexplained variation can be due to several factors. First of all, a high proportion of unexplained variation is typical for this type of multivariate analyses [69]. Not all settlements will have been detected and not all environmental variables that are of potential concern such as the proximity of important ancient sources of minerals such as clay or obsidian could be quantified [60]. Secondly, the relatively low density of human populations at that time ensures that a lot of areas which are suitable for settlement remained unsettled. As such, in terms of potential settlement, the dataset is highly unsaturated with a disproportionately large amount of empty cells and a very small amount of occupied cells. Therefore it is striking that despite a relatively small signal/noise ratio important and generally highly statistically significant patterns emerge which may reflect different deterministic drivers of human settlement. Finally, besides noise, the unexplained fraction also includes all variation generated by stochastic processes as well as rational motivations of people independent of the spatial proxies for responsible processes considered in this study.

### Perspectives

Overall, this study illustrates how an integration of historical, local and regional processes can contribute to a better understanding of patterns of human settlements and may generate novel hypotheses. Specifically for the studied region, this study showed that, although there are some changes, the dominant drivers of human settlement implicated by the studied correlates have remained much the same during the studied periods. One could argue that the inclusion of large numbers of MEM gives a large weight to space in models. However, since this issue is largely resolved due to the use the use of adjusted r square values [25], the method is suitable for comparative analyses. As such, if applied correctly, MEM based variation partitioning provides a powerful tool for comparative analyses among different regions and even for large scale meta-analyses covering many datasets [69]. The MEM approach allows to detect presence of spatial structure in their datasets and identify relevant spatial scales, enabling researchers to identify whether these can be attributed to environmental filtering or environment independent processes related to migration and history. Nonetheless, particularly explaining patterns of clustering independent of environmental conditions in terms of responsible processes remains an important challenge.

## Supporting Information

Figure S1 **Examples of Moran’s eigenvector maps corresponding to different spatial scales.** Overview of 10 Moran’s eigenvector maps (MEM’s) illustrating the increasingly smaller spatial scales described by MEM’s with increasing ranks. Red represents positive peaks of the spatial wave functions, while blue corresponds to negative dips.
(TIF)

Table S1 **Overview of the different trace artefacts indicative for the presence of humans during certain time periods.**
(DOCX)

Table S2 **Overview of environmental variables included in redundancy analysis models.**
(DOCX)

Table S3 **Overview of different MEM variables retained in redundancy analysis models after forward selection for each of the seven considered time periods.** Numbers correspond with the rank (R) of each of the generated MEMs with a positive Moran’s I (range: 1 - 3264). λ corresponds to the wavelength of each MEM expressed in km. Smaller wavelengths correspond to increasingly smaller scales of spatial clustering.
(DOCX)
