# Supplementary figures and images for: Disentangling the Spatio-Environmental Drivers of Human Settlement: An Eigenvector Based Variation Decomposition
Source: PLoS One. 2013 Jul 2;8(7):e67726. doi: 10.1371/journal.pone.0067726 (PMC3699633; doi:10.1371/journal.pone.0067726)

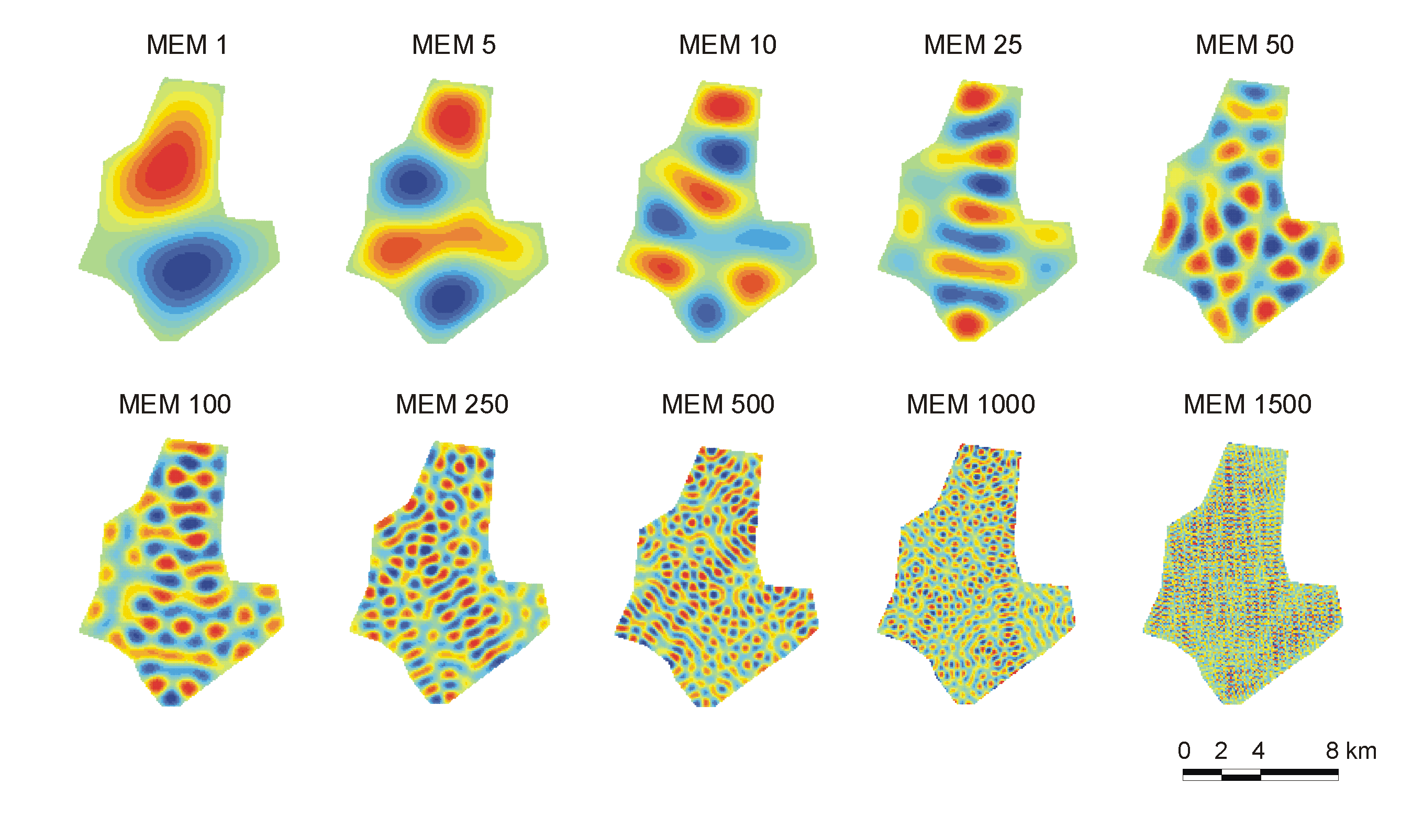

Supplement: Figure S1 — Examples of Moran’s eigenvector maps corresponding to different spatial scales. Overview of 10 Moran’s eigenvector maps (MEM’s) illustrating the increasingly smaller spatial scales described by MEM’s with increasing ranks. Red represents positive peaks of the spatial wave functions, while blue corresponds to negative dips. (TIF) [file pone.0067726.s001.tif]
