# Supplementary material for: Disentangling the Spatio-Environmental Drivers of Human Settlement: An Eigenvector Based Variation Decomposition
Source: PLoS One. 2013 Jul 2;8(7):e67726. doi: 10.1371/journal.pone.0067726 (PMC3699633; doi:10.1371/journal.pone.0067726)
Supplement: Table S1 — Overview of the different trace artefacts indicative for the presence of humans during certain time periods. (DOCX) [file pone.0067726.s002.docx]

|  |  |  |  |
| --- | --- | --- | --- |
| **Code** | **Period** | **Age** | **Unique pottery** |
| NEO_ECH | Late Neolithic/Early Chalcolithic | 6500-5500 BC | Handmade pottery, monochrome pottery with red-brown slip, painted pottery: red on cream, carinated bowls, tubular lug handles |
| LCH_EBI | Late Chalcolithic/Early Bronze Age I | 4000- 2600 BC | Handmade pottery: mMottled pottery, dark burnished pottery, straw tempered pottery, large open vessel types, ledge handles |
| EBII | Early Bronze Age II | 2600-2300 BC | Handmade pottery : gGrooved and ribbed decoration, red slipped pottery, spouted jugs, bowls with lug handles on the rim, cups with handles rising above rim, introduction of Depas Amphikypellon |
| A-CH | Archaic-Classical Hellenistic | 700-200 BC | Combination of coarse wares + decorated fine wares (Black-on-red ware, bichrome ware, matt painted ware)  Majority local production, but imports do occur occasionally |
| HELL | Hellenistic | 333-25 BC | More standardized repertoire over wider region  Increasing regional homogeneity  Reddish/brownish slipped mould made bowls  Black-glazed vessels  Uguentaria, fishplates |
| BYZ | Byzantine | 610-1300 AD | Rural production is dominant. Small number import finewares. Monochrome glazed wares, incised sgraffito, pattern burnished, Champlevé, slip painted |
