# Supplementary material for: Disentangling the Spatio-Environmental Drivers of Human Settlement: An Eigenvector Based Variation Decomposition
Source: PLoS One. 2013 Jul 2;8(7):e67726. doi: 10.1371/journal.pone.0067726 (PMC3699633; doi:10.1371/journal.pone.0067726)
Supplement: Table S2 — Overview of environmental variables included in redundancy analysis models. (DOCX) [file pone.0067726.s003.docx]

| **Code** | **Explanation** |
| --- | --- |
|  |  |
| ELEVATION: | altitude above sea level (m) |
| NEAR_RIVER: | distance to the nearest river (m) |
| NEAR_SPRNG: | distance to the nearest spring (m) |
| NEAR_HILL: | distance to the nearest hill (m) |
| P_BADLAND1: | % 'badland' in a radius of 1 km |
| P_HILL1: | % 'hill' in a radius of 1 km |
| P_LAKE1: | % 'lake' in a radius of 1 km |
| P_SWAMP1: | % 'swamp' in a radius of 1 km |
| P_VALLEY1: | % 'valley' in a radius of 1 km |
| P_BADLAND4: | % 'badland' in a radius of 4 km |
| P_HILL4: | % 'hill' in a radius of 4 km |
| P_LAKE4: | % 'lake' in a radius of 4 km |
| P_SWAMP4: | % 'swamp' in a radius of 4 km |
| P_VALLEY4: | % 'valley' in a radius of 4 km |
| VIEWSHED_1.4_1: | visible area (km²) for an average observer |
