# Supplementary material for: Disentangling the Spatio-Environmental Drivers of Human Settlement: An Eigenvector Based Variation Decomposition
Source: PLoS One. 2013 Jul 2;8(7):e67726. doi: 10.1371/journal.pone.0067726 (PMC3699633; doi:10.1371/journal.pone.0067726)
Supplement: Table S3 — Overview of different MEM variables retained in redundancy analysis models after forward selection for each of the seven considered time periods. Numbers correspond with the rank (R) of each of the generated MEMs with a positive Moran’s I (range: 1 - 3264). λ corresponds to the wavelength of each MEM expressed in km. Smaller wavelengths correspond to increasingly smaller scales of spatial clustering. (DOCX) [file pone.0067726.s004.docx]

|  |  |  |  |  |  |  |  |  |  |  |  |  |  |  |  |  |  |  |  |  |  |
| --- | --- | --- | --- | --- | --- | --- | --- | --- | --- | --- | --- | --- | --- | --- | --- | --- | --- | --- | --- | --- | --- |
| NEOL_ECHAL | | | | | |  | LCHAL_EBI | | | | | |  | EBII | | | | | | | |
| MEM | λ (km) | MEM | λ (km) | MEM | λ (km) |  | MEM | λ (km) | MEM | λ (km) | MEM | λ (km) |  | MEM | λ (km) | MEM | λ (km) | MEM | λ (km) | MEM | λ (km) |
| 3 | 44.9 | 247 | 0.72 | 969 | 0.19 |  | 1 | 89.9 | 102 | 1.75 | 311 | 0.58 |  | 3 | 44.9 | 135 | 1.32 | 277 | 0.65 | 604 | 0.30 |
| 5 | 30.0 | 255 | 0.70 | 985 | 0.18 |  | 2 | 59.9 | 106 | 1.68 | 313 | 0.57 |  | 5 | 30.0 | 136 | 1.31 | 279 | 0.64 | 614 | 0.29 |
| 6 | 25.7 | 259 | 0.69 | 1026 | 0.18 |  | 3 | 44.9 | 110 | 1.62 | 317 | 0.57 |  | 6 | 25.7 | 138 | 1.29 | 280 | 0.64 | 629 | 0.29 |
| 10 | 16.3 | 264 | 0.68 | 1076 | 0.17 |  | 4 | 36.0 | 112 | 1.59 | 321 | 0.56 |  | 7 | 22.5 | 141 | 1.27 | 285 | 0.63 | 633 | 0.28 |
| 12 | 13.8 | 266 | 0.67 | 1115 | 0.16 |  | 5 | 30.0 | 113 | 1.58 | 363 | 0.49 |  | 11 | 15.0 | 143 | 1.25 | 290 | 0.62 | 642 | 0.28 |
| 13 | 12.8 | 267 | 0.67 | 1116 | 0.16 |  | 6 | 25.7 | 116 | 1.54 | 381 | 0.47 |  | 17 | 10.0 | 150 | 1.19 | 291 | 0.62 | 653 | 0.27 |
| 18 | 9.5 | 279 | 0.64 | 1118 | 0.16 |  | 7 | 22.5 | 119 | 1.50 | 419 | 0.43 |  | 18 | 9.5 | 157 | 1.14 | 298 | 0.60 | 655 | 0.27 |
| 19 | 9.0 | 281 | 0.64 |  |  |  | 10 | 16.3 | 126 | 1.42 | 432 | 0.42 |  | 19 | 9.0 | 158 | 1.13 | 301 | 0.60 | 670 | 0.27 |
| 20 | 8.6 | 286 | 0.63 |  |  |  | 11 | 15.0 | 128 | 1.39 | 445 | 0.40 |  | 20 | 8.6 | 159 | 1.12 | 302 | 0.59 | 693 | 0.26 |
| 26 | 6.7 | 330 | 0.54 |  |  |  | 13 | 12.8 | 132 | 1.35 | 447 | 0.40 |  | 22 | 7.8 | 161 | 1.11 | 303 | 0.59 | 719 | 0.25 |
| 29 | 6.0 | 351 | 0.51 |  |  |  | 16 | 10.6 | 135 | 1.32 | 449 | 0.40 |  | 26 | 6.7 | 163 | 1.10 | 308 | 0.58 | 752 | 0.24 |
| 30 | 5.8 | 390 | 0.46 |  |  |  | 17 | 10.0 | 141 | 1.27 | 454 | 0.40 |  | 29 | 6.0 | 168 | 1.06 | 315 | 0.57 | 759 | 0.24 |
| 51 | 3.5 | 408 | 0.44 |  |  |  | 18 | 9.5 | 143 | 1.25 | 457 | 0.39 |  | 31 | 5.6 | 169 | 1.06 | 316 | 0.57 | 769 | 0.23 |
| 66 | 2.7 | 415 | 0.43 |  |  |  | 20 | 8.6 | 144 | 1.24 | 469 | 0.38 |  | 32 | 5.4 | 170 | 1.05 | 317 | 0.57 | 772 | 0.23 |
| 76 | 2.3 | 424 | 0.42 |  |  |  | 22 | 7.8 | 145 | 1.23 | 470 | 0.38 |  | 34 | 5.1 | 172 | 1.04 | 321 | 0.56 | 774 | 0.23 |
| 78 | 2.3 | 431 | 0.42 |  |  |  | 23 | 7.5 | 150 | 1.19 | 471 | 0.38 |  | 41 | 4.3 | 175 | 1.02 | 325 | 0.55 | 800 | 0.22 |
| 89 | 2.0 | 434 | 0.41 |  |  |  | 24 | 7.2 | 151 | 1.18 | 476 | 0.38 |  | 42 | 4.2 | 178 | 1.00 | 330 | 0.54 | 820 | 0.22 |
| 94 | 1.9 | 446 | 0.40 |  |  |  | 26 | 6.7 | 158 | 1.13 | 478 | 0.38 |  | 46 | 3.8 | 179 | 1.00 | 334 | 0.54 | 898 | 0.20 |
| 95 | 1.9 | 453 | 0.40 |  |  |  | 28 | 6.2 | 177 | 1.01 | 479 | 0.37 |  | 47 | 3.7 | 182 | 0.98 | 336 | 0.53 | 925 | 0.19 |
| 96 | 1.9 | 454 | 0.40 |  |  |  | 31 | 5.6 | 180 | 0.99 | 483 | 0.37 |  | 48 | 3.7 | 184 | 0.97 | 343 | 0.52 | 1190 | 0.15 |
| 99 | 1.8 | 455 | 0.39 |  |  |  | 32 | 5.4 | 189 | 0.95 | 506 | 0.35 |  | 50 | 3.5 | 186 | 0.96 | 350 | 0.51 |  |  |
| 106 | 1.7 | 475 | 0.38 |  |  |  | 33 | 5.3 | 191 | 0.94 | 509 | 0.35 |  | 51 | 3.5 | 187 | 0.96 | 351 | 0.51 |  |  |
| 117 | 1.5 | 478 | 0.38 |  |  |  | 35 | 5.0 | 199 | 0.90 | 535 | 0.34 |  | 53 | 3.3 | 189 | 0.95 | 358 | 0.50 |  |  |
| 125 | 1.4 | 484 | 0.37 |  |  |  | 39 | 4.5 | 202 | 0.89 | 539 | 0.33 |  | 54 | 3.3 | 191 | 0.94 | 364 | 0.49 |  |  |
| 126 | 1.4 | 493 | 0.36 |  |  |  | 42 | 4.2 | 206 | 0.87 | 551 | 0.33 |  | 60 | 2.9 | 194 | 0.92 | 367 | 0.49 |  |  |
| 130 | 1.4 | 506 | 0.35 |  |  |  | 43 | 4.1 | 209 | 0.86 | 565 | 0.32 |  | 62 | 2.9 | 196 | 0.91 | 370 | 0.48 |  |  |
| 136 | 1.3 | 510 | 0.35 |  |  |  | 45 | 3.9 | 217 | 0.82 | 573 | 0.31 |  | 65 | 2.7 | 197 | 0.91 | 372 | 0.48 |  |  |
| 143 | 1.2 | 527 | 0.34 |  |  |  | 48 | 3.7 | 218 | 0.82 | 579 | 0.31 |  | 67 | 2.6 | 199 | 0.90 | 380 | 0.47 |  |  |
| 147 | 1.2 | 536 | 0.33 |  |  |  | 50 | 3.5 | 227 | 0.79 | 586 | 0.31 |  | 69 | 2.6 | 200 | 0.89 | 381 | 0.47 |  |  |
| 148 | 1.2 | 540 | 0.33 |  |  |  | 52 | 3.4 | 230 | 0.78 | 610 | 0.29 |  | 71 | 2.5 | 205 | 0.87 | 384 | 0.47 |  |  |
| 150 | 1.2 | 562 | 0.32 |  |  |  | 55 | 3.2 | 231 | 0.77 | 611 | 0.29 |  | 73 | 2.4 | 208 | 0.86 | 393 | 0.46 |  |  |
| 155 | 1.2 | 563 | 0.32 |  |  |  | 57 | 3.1 | 232 | 0.77 | 670 | 0.27 |  | 74 | 2.4 | 212 | 0.84 | 400 | 0.45 |  |  |
| 160 | 1.1 | 565 | 0.32 |  |  |  | 60 | 2.9 | 234 | 0.76 | 705 | 0.25 |  | 75 | 2.4 | 213 | 0.84 | 401 | 0.45 |  |  |
| 168 | 1.1 | 566 | 0.32 |  |  |  | 61 | 2.9 | 235 | 0.76 | 715 | 0.25 |  | 78 | 2.3 | 223 | 0.80 | 418 | 0.43 |  |  |
| 170 | 1.1 | 588 | 0.31 |  |  |  | 63 | 2.8 | 245 | 0.73 | 820 | 0.22 |  | 82 | 2.2 | 226 | 0.79 | 428 | 0.42 |  |  |
| 172 | 1.0 | 591 | 0.30 |  |  |  | 65 | 2.7 | 249 | 0.72 | 916 | 0.20 |  | 87 | 2.0 | 231 | 0.77 | 432 | 0.42 |  |  |
| 175 | 1.0 | 599 | 0.30 |  |  |  | 71 | 2.5 | 252 | 0.71 | 1037 | 0.17 |  | 88 | 2.0 | 232 | 0.77 | 437 | 0.41 |  |  |
| 177 | 1.0 | 629 | 0.29 |  |  |  | 72 | 2.5 | 253 | 0.71 | 1060 | 0.17 |  | 89 | 2.0 | 233 | 0.77 | 439 | 0.41 |  |  |
| 180 | 1.0 | 662 | 0.27 |  |  |  | 73 | 2.4 | 256 | 0.70 | 1120 | 0.16 |  | 90 | 2.0 | 235 | 0.76 | 440 | 0.41 |  |  |
| 187 | 1.0 | 699 | 0.26 |  |  |  | 75 | 2.4 | 259 | 0.69 |  |  |  | 92 | 1.9 | 239 | 0.75 | 451 | 0.40 |  |  |
| 195 | 0.9 | 725 | 0.25 |  |  |  | 76 | 2.3 | 271 | 0.66 |  |  |  | 96 | 1.9 | 242 | 0.74 | 475 | 0.38 |  |  |
| 197 | 0.9 | 730 | 0.25 |  |  |  | 78 | 2.3 | 273 | 0.66 |  |  |  | 100 | 1.8 | 243 | 0.74 | 478 | 0.38 |  |  |
| 198 | 0.9 | 732 | 0.25 |  |  |  | 79 | 2.2 | 274 | 0.65 |  |  |  | 105 | 1.7 | 245 | 0.73 | 483 | 0.37 |  |  |
| 206 | 0.9 | 739 | 0.24 |  |  |  | 80 | 2.2 | 279 | 0.64 |  |  |  | 108 | 1.6 | 246 | 0.73 | 497 | 0.36 |  |  |
| 210 | 0.9 | 756 | 0.24 |  |  |  | 82 | 2.2 | 281 | 0.64 |  |  |  | 110 | 1.6 | 250 | 0.72 | 500 | 0.36 |  |  |
| 218 | 0.8 | 780 | 0.23 |  |  |  | 88 | 2.0 | 282 | 0.64 |  |  |  | 113 | 1.6 | 252 | 0.71 | 508 | 0.35 |  |  |
| 220 | 0.8 | 797 | 0.23 |  |  |  | 91 | 2.0 | 283 | 0.63 |  |  |  | 114 | 1.6 | 258 | 0.69 | 513 | 0.35 |  |  |
| 222 | 0.8 | 809 | 0.22 |  |  |  | 93 | 1.9 | 290 | 0.62 |  |  |  | 119 | 1.5 | 261 | 0.69 | 534 | 0.34 |  |  |
| 226 | 0.8 | 813 | 0.22 |  |  |  | 94 | 1.9 | 294 | 0.61 |  |  |  | 121 | 1.5 | 262 | 0.68 | 545 | 0.33 |  |  |
| 234 | 0.8 | 873 | 0.21 |  |  |  | 96 | 1.9 | 297 | 0.60 |  |  |  | 123 | 1.4 | 266 | 0.67 | 548 | 0.33 |  |  |
| 245 | 0.7 | 899 | 0.20 |  |  |  | 99 | 1.8 | 299 | 0.60 |  |  |  | 130 | 1.4 | 271 | 0.66 | 551 | 0.33 |  |  |
| 246 | 0.7 | 932 | 0.19 |  |  |  | 100 | 1.8 | 300 | 0.60 |  |  |  | 133 | 1.3 | 274 | 0.65 | 579 | 0.31 |  |  |
| Mean | 2.52 |  |  |  |  |  |  | 4.1 |  |  |  |  |  |  | 2.2 |  |  |  |  |  |  |
| SD | 6.22 |  |  |  |  |  |  | 10.7 |  |  |  |  |  |  | 5.0 |  |  |  |  |  |  |

|  |  |  |  |  |  |  |  |  |  |  |  |  |  |  |  |  |  |  |  |
| --- | --- | --- | --- | --- | --- | --- | --- | --- | --- | --- | --- | --- | --- | --- | --- | --- | --- | --- | --- |
| A-CH | | | | | |  | HELL | | | | | |  | BYZ | | | | | |
| MEM | λ (km) | MEM | λ (km) | MEM | λ (km) |  | MEM | λ (km) | MEM | λ (km) | MEM | λ (km) |  | MEM | λ (km) | MEM | λ (km) | MEM | λ (km) |
| 1 | 89.9 | 103 | 1.73 | 292 | 0.61 |  | 1 | 89.9 | 125 | 1.43 | 422 | 0.42 |  | 1 | 89.9 | 180 | 0.99 | 525 | 0.34 |
| 2 | 59.9 | 104 | 1.71 | 295 | 0.61 |  | 3 | 44.9 | 130 | 1.37 | 429 | 0.42 |  | 2 | 59.9 | 181 | 0.99 | 529 | 0.34 |
| 3 | 44.9 | 106 | 1.68 | 297 | 0.60 |  | 4 | 36.0 | 132 | 1.35 | 433 | 0.41 |  | 3 | 44.9 | 183 | 0.98 | 531 | 0.34 |
| 4 | 36.0 | 108 | 1.65 | 300 | 0.60 |  | 5 | 30.0 | 134 | 1.33 | 446 | 0.40 |  | 4 | 36.0 | 190 | 0.94 | 574 | 0.31 |
| 5 | 30.0 | 109 | 1.63 | 303 | 0.59 |  | 10 | 16.3 | 135 | 1.32 | 457 | 0.39 |  | 5 | 30.0 | 198 | 0.90 | 595 | 0.30 |
| 8 | 20.0 | 112 | 1.59 | 311 | 0.58 |  | 11 | 15.0 | 137 | 1.30 | 465 | 0.39 |  | 8 | 20.0 | 200 | 0.89 | 602 | 0.30 |
| 9 | 18.0 | 113 | 1.58 | 315 | 0.57 |  | 16 | 10.6 | 138 | 1.29 | 474 | 0.38 |  | 9 | 18.0 | 204 | 0.88 | 612 | 0.29 |
| 10 | 16.3 | 119 | 1.50 | 316 | 0.57 |  | 17 | 10.0 | 141 | 1.27 | 482 | 0.37 |  | 10 | 16.3 | 213 | 0.84 | 631 | 0.28 |
| 11 | 15.0 | 122 | 1.46 | 324 | 0.55 |  | 18 | 9.5 | 145 | 1.23 | 501 | 0.36 |  | 12 | 13.8 | 214 | 0.84 | 640 | 0.28 |
| 12 | 13.8 | 123 | 1.45 | 329 | 0.54 |  | 19 | 9.0 | 146 | 1.22 | 519 | 0.35 |  | 13 | 12.8 | 215 | 0.83 | 641 | 0.28 |
| 13 | 12.8 | 125 | 1.43 | 334 | 0.54 |  | 20 | 8.6 | 158 | 1.13 | 560 | 0.32 |  | 19 | 9.0 | 220 | 0.81 | 646 | 0.28 |
| 14 | 12.0 | 128 | 1.39 | 335 | 0.54 |  | 23 | 7.5 | 161 | 1.11 | 565 | 0.32 |  | 20 | 8.6 | 224 | 0.80 | 650 | 0.28 |
| 15 | 11.2 | 134 | 1.33 | 341 | 0.53 |  | 24 | 7.2 | 163 | 1.10 | 567 | 0.32 |  | 21 | 8.2 | 229 | 0.78 | 654 | 0.27 |
| 17 | 10.0 | 136 | 1.31 | 346 | 0.52 |  | 26 | 6.7 | 166 | 1.08 | 573 | 0.31 |  | 26 | 6.7 | 233 | 0.77 | 658 | 0.27 |
| 19 | 9.0 | 137 | 1.30 | 358 | 0.50 |  | 28 | 6.2 | 174 | 1.03 | 575 | 0.31 |  | 30 | 5.8 | 235 | 0.76 | 675 | 0.27 |
| 22 | 7.8 | 141 | 1.27 | 362 | 0.50 |  | 33 | 5.3 | 176 | 1.02 | 584 | 0.31 |  | 36 | 4.9 | 242 | 0.74 | 686 | 0.26 |
| 23 | 7.5 | 145 | 1.23 | 363 | 0.49 |  | 34 | 5.1 | 187 | 0.96 | 599 | 0.30 |  | 38 | 4.6 | 248 | 0.72 | 704 | 0.25 |
| 24 | 7.2 | 147 | 1.21 | 374 | 0.48 |  | 38 | 4.6 | 190 | 0.94 | 614 | 0.29 |  | 40 | 4.4 | 258 | 0.69 | 711 | 0.25 |
| 26 | 6.7 | 149 | 1.20 | 375 | 0.48 |  | 39 | 4.5 | 195 | 0.92 | 620 | 0.29 |  | 44 | 4.0 | 266 | 0.67 | 713 | 0.25 |
| 27 | 6.4 | 150 | 1.19 | 378 | 0.47 |  | 41 | 4.3 | 199 | 0.90 | 632 | 0.28 |  | 48 | 3.7 | 269 | 0.67 | 881 | 0.20 |
| 29 | 6.0 | 157 | 1.14 | 393 | 0.46 |  | 48 | 3.7 | 207 | 0.86 | 647 | 0.28 |  | 50 | 3.5 | 270 | 0.66 | 887 | 0.20 |
| 30 | 5.8 | 159 | 1.12 | 406 | 0.44 |  | 51 | 3.5 | 208 | 0.86 | 654 | 0.27 |  | 52 | 3.4 | 273 | 0.66 | 966 | 0.19 |
| 31 | 5.6 | 163 | 1.10 | 412 | 0.44 |  | 52 | 3.4 | 212 | 0.84 | 683 | 0.26 |  | 59 | 3.0 | 274 | 0.65 | 978 | 0.18 |
| 34 | 5.1 | 166 | 1.08 | 422 | 0.42 |  | 58 | 3.0 | 214 | 0.84 | 695 | 0.26 |  | 60 | 2.9 | 278 | 0.64 | 984 | 0.18 |
| 36 | 4.9 | 169 | 1.06 | 433 | 0.41 |  | 59 | 3.0 | 219 | 0.82 | 699 | 0.26 |  | 63 | 2.8 | 282 | 0.64 | 993 | 0.18 |
| 38 | 4.6 | 171 | 1.05 | 446 | 0.40 |  | 61 | 2.9 | 223 | 0.80 | 759 | 0.24 |  | 69 | 2.6 | 283 | 0.63 | 1019 | 0.18 |
| 41 | 4.3 | 172 | 1.04 | 461 | 0.39 |  | 63 | 2.8 | 224 | 0.80 | 833 | 0.22 |  | 71 | 2.5 | 287 | 0.62 | 1053 | 0.17 |
| 44 | 4.0 | 174 | 1.03 | 469 | 0.38 |  | 65 | 2.7 | 227 | 0.79 | 849 | 0.21 |  | 72 | 2.5 | 290 | 0.62 | 1063 | 0.17 |
| 47 | 3.7 | 175 | 1.02 | 497 | 0.36 |  | 69 | 2.6 | 233 | 0.77 | 1072 | 0.17 |  | 74 | 2.4 | 294 | 0.61 | 1094 | 0.16 |
| 48 | 3.7 | 178 | 1.00 | 524 | 0.34 |  | 74 | 2.4 | 235 | 0.76 | 1174 | 0.15 |  | 76 | 2.3 | 300 | 0.60 | 1150 | 0.16 |
| 51 | 3.5 | 180 | 0.99 | 537 | 0.33 |  | 78 | 2.3 | 238 | 0.75 | 1206 | 0.15 |  | 78 | 2.3 | 316 | 0.57 |  |  |
| 52 | 3.4 | 181 | 0.99 | 563 | 0.32 |  | 79 | 2.2 | 241 | 0.74 | 1209 | 0.15 |  | 80 | 2.2 | 322 | 0.56 |  |  |
| 53 | 3.3 | 184 | 0.97 | 594 | 0.30 |  | 80 | 2.2 | 256 | 0.70 |  |  |  | 81 | 2.2 | 327 | 0.55 |  |  |
| 58 | 3.0 | 195 | 0.92 | 598 | 0.30 |  | 83 | 2.1 | 267 | 0.67 |  |  |  | 83 | 2.1 | 346 | 0.52 |  |  |
| 59 | 3.0 | 198 | 0.90 | 653 | 0.27 |  | 88 | 2.0 | 271 | 0.66 |  |  |  | 84 | 2.1 | 347 | 0.52 |  |  |
| 63 | 2.8 | 199 | 0.90 | 672 | 0.27 |  | 89 | 2.0 | 274 | 0.65 |  |  |  | 89 | 2.0 | 355 | 0.50 |  |  |
| 65 | 2.7 | 204 | 0.88 | 682 | 0.26 |  | 91 | 2.0 | 275 | 0.65 |  |  |  | 91 | 2.0 | 360 | 0.50 |  |  |
| 66 | 2.7 | 212 | 0.84 | 683 | 0.26 |  | 93 | 1.9 | 279 | 0.64 |  |  |  | 94 | 1.9 | 366 | 0.49 |  |  |
| 69 | 2.6 | 214 | 0.84 | 689 | 0.26 |  | 94 | 1.9 | 286 | 0.63 |  |  |  | 99 | 1.8 | 382 | 0.47 |  |  |
| 70 | 2.5 | 216 | 0.83 | 770 | 0.23 |  | 96 | 1.9 | 297 | 0.60 |  |  |  | 100 | 1.8 | 391 | 0.46 |  |  |
| 74 | 2.4 | 217 | 0.82 | 794 | 0.23 |  | 100 | 1.8 | 300 | 0.60 |  |  |  | 112 | 1.6 | 407 | 0.44 |  |  |
| 78 | 2.3 | 219 | 0.82 | 811 | 0.22 |  | 101 | 1.8 | 324 | 0.55 |  |  |  | 122 | 1.5 | 412 | 0.44 |  |  |
| 80 | 2.2 | 224 | 0.80 | 932 | 0.19 |  | 103 | 1.7 | 325 | 0.55 |  |  |  | 127 | 1.4 | 424 | 0.42 |  |  |
| 82 | 2.2 | 236 | 0.76 | 992 | 0.18 |  | 104 | 1.7 | 355 | 0.50 |  |  |  | 128 | 1.4 | 425 | 0.42 |  |  |
| 83 | 2.1 | 241 | 0.74 | 1033 | 0.17 |  | 106 | 1.7 | 356 | 0.50 |  |  |  | 141 | 1.3 | 429 | 0.42 |  |  |
| 85 | 2.1 | 256 | 0.70 | 1079 | 0.17 |  | 107 | 1.7 | 373 | 0.48 |  |  |  | 142 | 1.3 | 438 | 0.41 |  |  |
| 89 | 2.0 | 264 | 0.68 | 1155 | 0.16 |  | 108 | 1.6 | 374 | 0.48 |  |  |  | 147 | 1.2 | 471 | 0.38 |  |  |
| 91 | 2.0 | 271 | 0.66 |  |  |  | 109 | 1.6 | 375 | 0.48 |  |  |  | 149 | 1.2 | 479 | 0.37 |  |  |
| 93 | 1.9 | 274 | 0.65 |  |  |  | 112 | 1.6 | 384 | 0.47 |  |  |  | 154 | 1.2 | 493 | 0.36 |  |  |
| 94 | 1.9 | 275 | 0.65 |  |  |  | 113 | 1.6 | 390 | 0.46 |  |  |  | 156 | 1.1 | 497 | 0.36 |  |  |
| 96 | 1.9 | 279 | 0.64 |  |  |  | 119 | 1.5 | 407 | 0.44 |  |  |  | 159 | 1.1 | 514 | 0.35 |  |  |
| 101 | 1.8 | 286 | 0.63 |  |  |  | 120 | 1.5 | 412 | 0.44 |  |  |  | 177 | 1.0 | 519 | 0.35 |  |  |
| Mean | 4.1 |  |  |  |  |  |  | 3.3 |  |  |  |  |  |  | 3.8 |  |  |  |  |
| SD | 10.4 |  |  |  |  |  |  | 9.5 |  |  |  |  |  |  | 10.9 |  |  |  |  |
